# Circumventing Zygotic Lethality to Generate Maternal Mutants in Zebrafish

**DOI:** 10.3390/biology11010102

**Published:** 2022-01-10

**Authors:** De-Li Shi

**Affiliations:** 1 Affiliated Hospital of Guangdong Medical University, Zhanjiang 524001, China; de-li.shi@upmc.fr; 2 Laboratory of Developmental Biology, CNRS-UMR7622, Institut de Biologie Paris-Seine, Sorbonne University, 75005 Paris, France

**Keywords:** maternal-effect genes, maternal mutants, zygotic lethality, CRISPR/Cas9, genome editing, transgenesis, oocyte-specific conditional knockout, zebrafish

## Abstract

**Simple Summary:**

Maternal gene products accumulated during oogenesis as mRNAs and proteins, which are referred to as maternal contributions, play essential roles in proper development before and after the transcription of the zygotic genome. Zebrafish represent an attractive model for the systematic characterization of genetic mutants using the CRISPR/Cas9 genome editing technology. The traditional approach for generating maternal mutants to study the functions of maternal gene products consists of creating founder fish with germ-line transmission of mutated genes, followed by successive crossing to obtain zygotic homozygous mutant females, which can produce offspring lacking the corresponding maternal gene products. This is time-consuming and normally requires three generations. Nevertheless, zygotic mutations of many genes often lead to embryonic lethality or adult sterility, making it impossible to study their maternal functions. Different approaches were used to overcome this difficulty and allowed for the characterization of several important maternal-effect genes. However, they are often technically challenging or lack versatile applications. Recently, an oocyte-specific knockout strategy has been developed, which inactivates genes of interest in the developing oocytes. It is particularly accessible, generating maternal mutants in one fish generation. By further improving its efficiency, this method can be used for the large-scale analysis of maternal gene functions.

**Abstract:**

Maternal gene products accumulated during oogenesis are essential for supporting early developmental processes in both invertebrates and vertebrates. Therefore, understanding their regulatory functions should provide insights into the maternal control of embryogenesis. The CRISPR/Cas9 genome editing technology has provided a powerful tool for creating genetic mutations to study gene functions and developing disease models to identify new therapeutics. However, many maternal genes are also essential after zygotic genome activation; as a result, loss of their zygotic functions often leads to lethality or sterility, thus preventing the generation of maternal mutants by classical crossing between zygotic homozygous mutant adult animals. Although several approaches, such as the rescue of mutant phenotypes through an injection of the wild-type mRNA, germ-line replacement, and the generation of genetically mosaic females, have been developed to overcome this difficulty, they are often technically challenging and time-consuming or inappropriate for many genes that are essential for late developmental events or for germ-line formation. Recently, a method based on the oocyte transgenic expression of CRISPR/Cas9 and guide RNAs has been designed to eliminate maternal gene products in zebrafish. This approach introduces several tandem guide RNA expression cassettes and a GFP reporter into transgenic embryos expressing Cas9 to create biallelic mutations and inactivate genes of interest specifically in the developing oocytes. It is particularly accessible and allows for the elimination of maternal gene products in one fish generation. By further improving its efficiency, this method can be used for the systematic characterization of maternal-effect genes.

## 1. Introduction

Maternal gene products accumulated during oogenesis as mRNAs and proteins make an important contribution to early development, such as egg polarity and activation, cytokinesis, germ layer and embryonic axis specification, morphogenetic cell movements, and organogenesis [1,2,3,4,5]. Maternally expressed genes represent a larger part of coding genes in zebrafish [6]. They not only play critical roles before zygotic genome activation but also perform essential functions well beyond this stage [3,7]. Zebrafish have become an important and attractive model for the large-scale mutational screens of gene functions, which is instrumental for understanding developmental mechanisms and illustrating the genome–phenome relationship [8,9,10], as well as for developing disease models and validating novel drug targets to identify new therapeutics [11,12]. As a simple and powerful tool for genome editing, the CRISPR/Cas9 technology has further promoted systematic functional analyses of developmental genes in this species [13,14]. The traditional approach for generating maternal mutants in forward and reverse genetics studies consists of creating germ-line transmissible F0 founders and successive crossing between mutant adults, which normally requires three generations (about 9 months) in zebrafish [8]. However, this can be prevented when the loss of zygotic gene functions leads to lethality or sterility. Several methods were used to circumvent this difficulty and allowed for the generation of maternal mutants for zygotic lethal genes. They mainly include the rescue of zygotic defective phenotypes by injecting the wild-type mRNA into fertilized eggs [15], germ-line replacement [16,17], oocyte microinjection in situ (OMIS) combined with gene knockout or knockdown [18], the generation of genetic mosaic females with biallelic mutations in the oocytes [19], conditional gene inactivation by the artificial chromosome-rescue-based knockout (BACK) approach [20], or the production of maternal crispants [21]. These methods helped to unravel the essential roles of a number of maternal-effect genes. However, several limitations, such as methods that are technically challenging, low-efficiency, time-consuming, or inappropriate for genes critically required for late developmental events or germ-line formation, restrict their use in the systematic analyses of maternal gene functions.

Recently, a novel strategy has been developed for the generation of maternal mutants and maternal-zygotic (MZ) mutants by circumventing zygotic lethality in a technically accessible, time-saving, and efficient manner [22,23]. It relies on the oocyte transgenic expression of CRISPR/Cas9 and guide RNAs (gRNAs) to target genes of interest and produces maternal mutants in the next generation (3 months). Thus, the oocyte-specific expression of multiple ribonucleoprotein complexes overcomes the difficulty of zygotic lethal effects for obtaining homozygous mutant adults. By further improving the efficiency, this oocyte-specific conditional knockout strategy should open the door for the systematic identification and characterization of maternal gene functions during early development. 

## 2. Strategies to Generate Maternal Mutants for Zygotic Lethal Genes in Zebrafish

### 2.1. Injection of the Wild-Type mRNA into Fertilized Eggs

**Figure 1:**
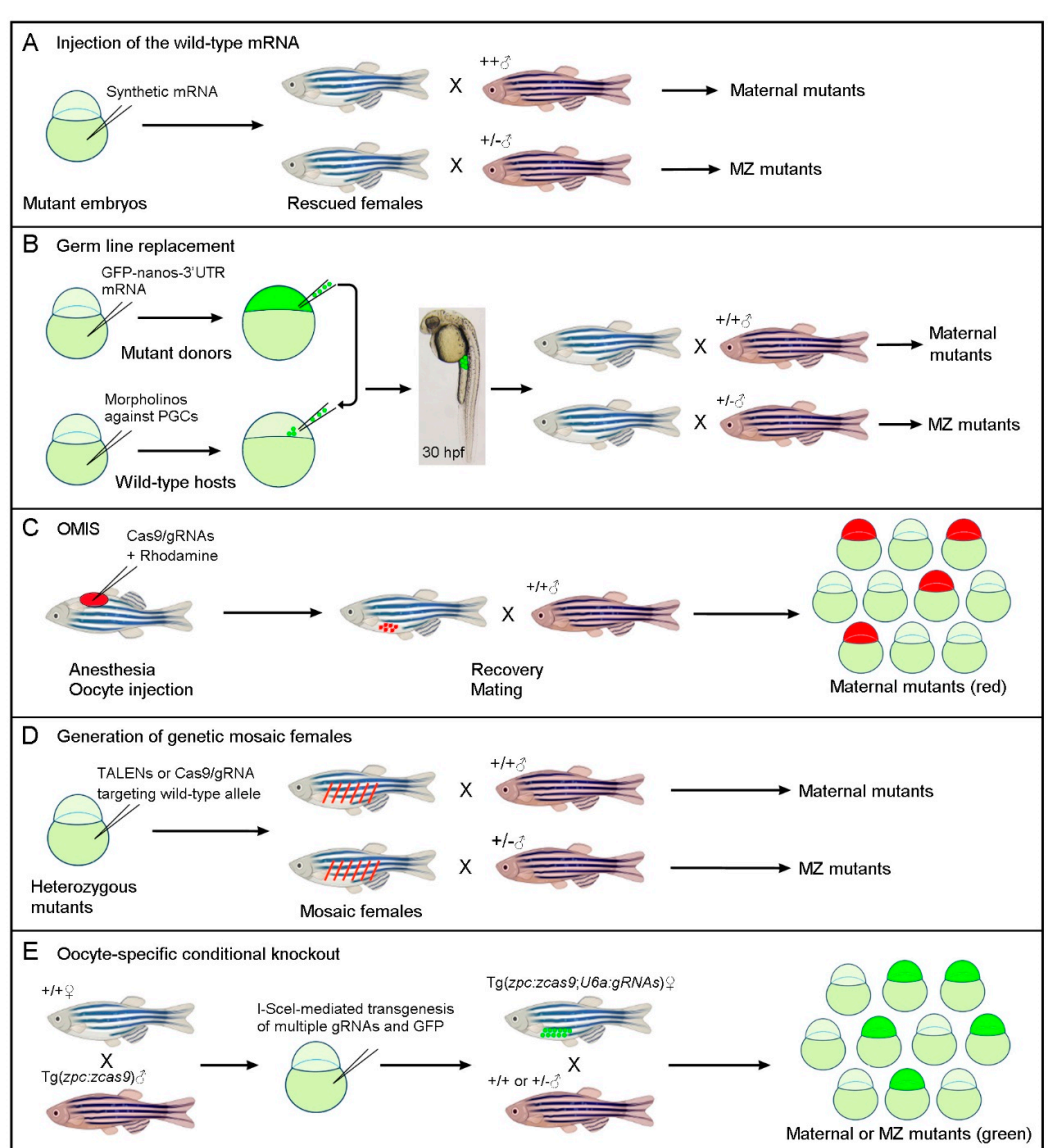
Simplified schematic representations of different strategies for generating maternal or MZ mutants by circumventing zygotic lethality. (**A**) Rescue of zygotic lethal phenotypes through the injection of the wild-type mRNA into fertilized homozygous mutant embryos. (**B**) Germ-line replacement through the transfer of zygotic lethal mutations to wild-type host embryos, in which PGCs are ablated by the morpholino-mediated inhibition of Dead-end protein function. Successful transplantation of fluorescently labeled PGCs can be monitored at 30 hpf (hours post-fertilization) by examining the presence of fluorescence in the gonadal mesoderm at the anterior region of the yolk extension. (**C**) The OMIS procedure generates maternal mutants in F0 embryos through the injection of Cas9/gRNAs in immature oocytes within the ovary. (**D**) Genetic mosaic females with biallelic mutations in the PGCs can be generated by targeting the wild-type allele in viable heterozygous mutant embryos. This mosaic approach can also be used to generate maternal crispants. (**E**) Oocyte-specific conditional knockout through the transgenic expression of Cas9 and multiple gRNAs targeting a gene of interest. According to the crosses, maternal or MZ mutants can be identified among GFP-positive offspring in the F1 generation.

For many maternally expressed genes, the loss of their zygotic functions causes severe phenotypes that often prevent survival either in the embryonic stages or in the adult. A simple and direct way to rescue the defective phenotypes but not the genotype is to inject in vitro synthesized wild-type mRNA into mutant embryos (Figure 1A). This was first successfully used in generating homozygous females for the *oep* null allele with the loss of the *EGF-CFC* gene that is critically required for germ layer formation and embryonic axis positioning during early development [15]. Since injected mRNAs and translated proteins are only stable for a short period (a few hours to a few days), this strategy may not be applied for maternal genes that also display essential functions at later stages of development.

### 2.2. Germ Line Replacement

In zebrafish, germ plasm ribonucleoparticles are first enriched in the vicinity of the cleavage furrows at the four-cell stage, and subsequently, primordial germ cells (PGCs) are located at the blastoderm margin of blastula stage embryos as four clusters [24]. For germ-line replacement, a group of 50–100 cells is removed from the margin of fluorescently labeled homozygous mutant donor embryos at the blastula stage. They are then transplanted into the blastoderm of host embryos in which only the formation of PGCs is prevented by antisense morpholino against the Dead-end RNA-binding protein essential for PGC survival (Figure 1B). This elegant technique was first used to generate maternal and MZ mutants for the zygotic lethal *miles apart* (*mil*) mutation that impairs cardiac precursor migration [16]. For general applications, its success depends not only on the transplantation of sufficient numbers of PGCs into the hosts but also on the efficient removal of host PGCs by morpholino-mediated knockdown. Since there are only four to eight PGCs located as four clusters at the blastoderm margin of blastula stage embryos [24], and reduced numbers of PGCs often lead to the masculinization of zebrafish [25], it is sometimes challenging to obtain adult females carrying transplanted mutant PGCs.

To increase the efficiency in PGC transplantation, a strategy that uses ectopically induced and Cas9/gRNA-targeted PGCs as donors, was reported recently [17]. It consists of co-injecting a gRNA targeting a gene of interest and a *cas9* coding region fused with the 3′-untranslated region (UTR) of *nanos* mRNA, which can stabilize and target Cas9 protein expression specifically in PGCs [26], along with the *bucky ball* (*buc*) mRNA that encodes a protein with activity to induce germ plasm formation [27]. MZ mutants for several embryonic lethal genes have been generated using this approach with relatively high efficiency [17]. 

### 2.3. Oocyte Microinjection In Situ

A straightforward procedure to obtain maternal mutants in F0 embryos is to perform genome editing directly in the immature oocytes within the ovary, thus preventing the accumulation of maternal gene products in fertilized eggs. This may be achieved by injecting Cas9/gRNAs into immature oocytes using the strategy of oocyte microinjection in situ (OMIS), so named because the manipulation of oocytes is realized in anesthetized adult zebrafish [18]. The oocytes subjected to genome editing are labeled with a cell lineage tracer, thus allowing for the identification of mutant embryos among the F0 offspring (Figure 1C). Through this procedure, stage III oocytes arrested at prophase I of meiosis were successfully edited before fertilization, and high efficiency biallelic mutations were obtained in single or multiple loci [18]. It is particularly time-saving for obtaining maternal mutants, but the in situ manipulation of immature oocytes may be technically challenging for beginners.

### 2.4. Generation of Genetic Mosaic Females

This approach was successfully used to generate double MZ mutants for *dishevelled 2* (*dvl2*) and *dishevelled 3a* (*dvl3a*), which participate in Wnt signaling and play predominant roles in embryonic patterning and morphogenetic movements [28]. Homozygous *dvl2* mutants are embryonically lethal, but *dvl2* and *dvl3a* triallelic mutants (heterozygous for *dvl2* and homozygous for *dvl3a*) are viable and fertile [19]. The strategy for generating genetic mosaic females was designed to further target the *dvl2* wild-type allele in triallelic mutant embryos by injecting TALENs (transcription activator-like effector nucleases) mRNAs or Cas9/gRNA into fertilized eggs, which are derived from crosses between triallelic carriers. Due to the mosaic distribution of injected mRNAs and incomplete targeting efficiency, the viable chimera with a proportion of PGCs carrying biallelic mutations in the *dvl2* loci (a new indel along with the original indel) could be obtained with relatively high efficiency. When crossed with triallelic mutant male fish, double MZ mutants for the two loci can be identified among the offspring through allele-specific PCR combined with Sanger sequencing (Figure 1D). This approach helped to decipher Dvl-mediated Wnt signaling pathways during early vertebrate development, especially the enigma of its implication in maternal Wnt/ß-catenin signaling. However, it is particularly time-consuming when manipulating two or more loci due to the necessity of genotyping a large number of carriers and offspring. 

### 2.5. Maternal Crispants

This approach is also based on the generation of genetic mosaic females and relies on the high rate of biallelic genome editing events in PGCs for the characterization of maternal-effect genes in F1 offspring [21]. To increase the efficiency, four gRNAs targeting a single gene of interest, along with Cas9 protein, were injected into fertilized eggs, which was shown to be more effective in generating biallelic mutations [29,30]. If viable embryos reach adulthood, the resulting females, when crossed with wild-type male fish, have the ability to produce offspring devoid of maternal products for the targeted gene. The proportion of F1 embryos with maternal-effect phenotypes (maternal crispants) may vary significantly, depending on the fraction of PGCs containing biallelic mutations [21]. Since this procedure also targets somatic cells, there is a possibility that high genome editing efficiency may produce F0 embryos with zygotic defective phenotypes (crispants) when the targeted gene is essential during late development.

### 2.6. Oocyte Transgenic Expression of CRISPR/Cas9 and gRNAs

An alternative to the above-mentioned methods is to achieve germ cell-specific conditional knockout. The BACK approach was developed to first rescue the homozygous mutant phenotype using a *loxP*-modified gene through Tol2-mediated transgenesis, and then perform conditional knockout through crossing between the rescued line and an established fish line with Cre expression specifically restricted in the PGCs [20]. This approach not only needs to create a mutant for the gene of interest, but also requires the generation of two independent transgenic lines. Thus, it is time-consuming and may not be appropriate for targeting those genes whose functions are indispensable for primordial cell development. 

A novel strategy that allows for the generation of maternal or MZ mutants in the absence of viable and fertile homozygous mutant adults and can potentially be applied in the functional screening of maternal factors was developed recently [22,23]. It takes advantage of the established transgenic line Tg(*zpc:zcas9*) that allows the specific expression of zebrafish codon-optimized *cas9* as early as in stage I oocytes under the control of the zona pellucida (*zpc*) gene promoter [31,32]. Genes of interest can be targeted through the U6 promoter-driven transgenic expression of gRNAs [33] in Cas9-expressing F0 zebrafish embryos. Through I-SceI meganuclease-mediated transgenesis [34], a single or multiple gRNA expression module, along with a GFP reporter, can be introduced into Tg(*zpc:zcas9*) embryos. Using this approach, founder fish with the conditional knockout of genes of interest in the developing oocytes can be produced effortlessly. When crossed with wild-type male fish, maternal mutants can be identified among GFP-positive F1 offspring with high efficiency. MZ mutants can be also obtained by just one generation through crossing between a female founder and a heterozygous male carrier (Figure 1E). This oocyte-specific conditional knockout strategy is technically accessible and presents the advantage that founder fish can be used repeatedly in their lifespan. Moreover, it can be used to simultaneously inactivate multiple maternal genes with functional redundancy [23], although in this case, the editing efficiency needs further improvement. 

## 3. Discussion

Genome editing technologies, particularly the CRISPR/Cas9 system, offer unprecedented opportunities for the study of gene functions during early development. Oocyte-stored mRNAs and proteins, referred to as maternal contributions, play critical roles before and after zygotic genome activation [1,2,3,4,5]. In zebrafish, and in other species such as *Xenopus*, the functions of a large number of maternal genes remain unexplored [35], mostly due to the bottleneck of efficiently eliminating maternally accumulated gene products. Significant efforts have been made to overcome the difficulty of embryonic lethality that prevents the generation of maternal and MZ mutants through the traditional approach, and the different strategies discussed here present both the advantages and limitations. The injection of the wild-type mRNA is certainly the most convenient manner of rescuing lethal phenotypes without the need for extensive genotyping [15], but this can only be applied for a small proportion of maternal genes with early functions while dispensable for late development. Nevertheless, if a specific lethal phenotype results from the dysfunction of a particular organ or tissue, it can be rescued by the transgenic expression of the wild-type sequence under the control of an appropriate promoter. Tol2-mediated transgenesis may be one of the options for this purpose [36]. The elegant procedure of germ-line replacement transfers zygotic lethal mutations through the germ-line [16], but its success is conditioned by the number of transplanted PGCs; thus, it is influenced by the sex bias. Therefore, increasing the efficiency of ectopically induced and Cas9/gRNA-targeted PGCs presents the potential to ensure the success of germ-line replacement [17]. OMIS is remarkably rapid for producing maternal mutants in F0 embryos [18], which may require strong expertise in manipulating immature oocytes within the ovary. The generation of genetic mosaic females and the subsequent analyses of maternal or MZ mutant offspring are both time-consuming and labor-extensive, although this approach did create germ-line transmissible biallelic mutations for multiple loci and helped to elucidate the important roles of maternal genes [19]. The approach of maternal crispants may represent an effective strategy for studying maternal-effect genes [21] if the high rate of biallelic mutations induces no defective phenotypes in the mosaic founders. 

In terms of technical accessibility and editing efficiency, the oocyte-specific conditional knockout approach through the transgenesis of Cas9 and multiple gRNAs targeting a single gene is promising to circumvent zygotic lethality, allowing for the generation of maternal or MZ mutants in F1 offspring. Importantly, it also presents a strong advantage for studying maternal functions of many genes whose zygotic mutations affect germ cell development. In this situation, the mutations can be neither transferred to host embryos through germ-line replacement nor transmitted to the next generation through genetic mosaic females. Also of note, the oocyte expression of Cas9 and multiple gRNAs was found to induce large deletions in the genome with high efficiency compared with genome editing in fertilized eggs [22,23]. This may facilitate the analyses of promoter regions and transcriptional or post-transcriptional elements. Nevertheless, the efficiency of this method also depends on the genome-editing activity of transgenically expressed Cas9 in the developing oocytes. Although the zebrafish codon-optimized *cas9* can mediate efficient genome editing with biallelic mutations [37,38], further improvement of its expression, stability, and translational efficiency in the oocytes of transgenic zebrafish may be necessary, such that it can be applied in the simultaneous editing of multiple maternal genes with functional redundancy. In this regard, the new RNA-guided endonuclease, Cas12a, possesses the ability to simplify multiplexed genome editing in various organisms or cell types [39]. Interestingly, it has recently been shown that engineered Cas12a could mediate simple and efficient genome editing in zebrafish, enabling its broad application for the functional analyses of maternal gene products through knockout and knockin approaches [40,41]. 

## 4. Conclusions

Different strategies, with both advantages and inconveniences, have been developed to circumvent zygotic lethality in the generation of maternal mutants. They helped to decipher the maternal functions of some zygotic lethal genes. However, the contribution of most maternal genes to early development remains largely elusive. In addition, many maternal genes exhibit functional redundancy, making it necessary to realize the simultaneous generation of multiple gene knockouts in order to understand mechanisms underlying maternal control of early development. Thus, the improvement of genome-editing technologies and the development of novel approaches for the rapid and efficient generation of maternal mutants will certainly facilitate the identification and functional characterization of maternal-effect genes.

## Data Availability

Not applicable.
